# Analysis of Immunosuppression and Antioxidant Damage in Diploid and Triploid Crucian Carp (*Carassius auratus*) Induced by Saline-Alkaline Environmental Stress: From Metabolomic Insight

**DOI:** 10.3390/metabo14120721

**Published:** 2024-12-21

**Authors:** Fangying Yuan, Xiaofeng Wei, Dongping Li, Xiaofeng Jin, Jing Wang, Yanchun Sun

**Affiliations:** 1 Laboratory of Quality & Safety Risk Assessment for Aquatic Products, Heilongjiang River Fisheries Research Institute, Chinese Academy of Fishery Sciences, Ministry of Agriculture and Rural Areas, Harbin 150070, China; yuanfy8013@163.com (F.Y.); jinxf_2023@163.com (X.J.); 2 Department of Chemical Engineering and Technology, College of Materials and Chemical Engineering, Harbin University of Science and Technology, Harbin 150080, China; lidongping@hrbust.edu.cn (D.L.); wangj199808@163.com (J.W.); 3 Department of Food Science and Engineering, College of Food Science and Engineering, Dalian Ocean University, Dalian 116023, China; 15234420692@163.com

**Keywords:** diploid crucian carp, triploid crucian carp, saline-alkaline stress, metabolites difference, serum metabolomics

## Abstract

**Objectives:** The salinization of the water environment worldwide is increasing, which has brought great challenges to the sustainability of fish farming of aquatic animals. **Methods:** Three NaHCO_3_ concentration groups (0 mmol/L, 20 mmol/L, and 60 mmol/L) were set up in this study to investigate growth and metabolic differences between diploid and triploid crucian carp under saline-alkaline stresses. **Purpose:** This study utilized UPLC-QTOF/MS metabolomics to analyze significant metabolites and metabolic pathways in the serum of diploid and triploid crucian carp, exposing them to different NaHCO_3_ concentrations in saline-alkaline habitats, elucidating the mechanism of their metabolic differences. **Results:** Results revealed that in the CA20 group, diploid and triploid crucian carp shared 69 differential metabolites, primarily enriched in pathways such as sphingolipid metabolism, glycerophospholipid metabolism, and linoleic acid metabolism. In the CA60 group, 46 differentially metabolites (DMs) were identified, mainly enriched in pathways such as linoleic acid metabolism, unsaturated fatty acid biosynthesis and sphingolipid metabolism. **Conclusions:** The analysis indicated that under different carbonate-saline-alkaline concentrations, diploid and triploid crucian carp primarily enriched in metabolic pathways such as glycerophospholipid metabolism, sphingolipid metabolism, and unsaturated fatty acid biosynthesis. With increasing carbonate-alkaline concentrations, hemolytic phospholipids associated with cell apoptosis were significantly upregulated and sphingolipid metabolism related to inflammation was more significantly enriched in triploid crucian carp, indicating that triploid crucian carp exhibited significant sensitivity to high carbonate-saline-alkaline stress and poorer carbonate-saline-alkaline tolerance. The results of this study provided a scientific theoretical basis for the later cultivation and aquaculture research of saline-alkaline-tolerant fish species.

## 1. Introduction

With the influence of human activities and climate change, among other factors, saline waters are becoming more widely distributed globally, with saline areas occupying about one third of the total land area. However, as the global demand for aquatic products continues to increase, freshwater resources are decreasing [1]. Therefore, the development and utilization of saline water resources for aquaculture has become a global challenge. China, a prominent freshwater aquaculture nation, faces formidable challenges in achieving sustainable development due to the limited potential for increased utilization of existing freshwater resources [2]. Saline-alkaline areas constitute a crucial component of the world’s land resources, with China alone accounting for approximately 4.6 million hectares of low-lying saline-alkaline land [3]. These saline-alkaline waters are extensively distributed in China’s inland regions, including the northwest, north, and northeast. Presently, saline-alkaline waters are classified into various types based on their chemical composition, with the carbonate type being the most extensive [4]. Saline-alkaline environments are characterized by high salinity, elevated pH, and an imbalanced ratio of major ions, resulting in extremely low biological productivity [5]. According to CNKI (China National Knowledge Infrastructure) and WOS (Web of Science) databases, the alkalinity of the majority of China’s saline-alkaline waters falls within the range of 8 to 30 mmol/L, and waters with alkalinity exceeding 15 mmol/L significantly impede the normal growth, development, and reproduction of fish, leading to prolonged states of biological inability to reproduce [6]. Consequently, the breeding of saline-alkaline tolerant fish varieties and the establishment of suitable aquaculture models are essential for transforming the extensive saline-alkaline water resources into valuable assets. This approach not only facilitates the economic reuse of fisheries but also addresses the scarcity of freshwater resources in the aquaculture sector [7]. It is also one of the key ways to efficiently utilize saline-alkaline waters.

Crucian carp (*Carassius auratus*), recognized as a significant species in large-scale aquaculture, is a bony fish adaptable to both freshwater and saline-alkaline environments [8]. Globally, the scale of crucian carp aquaculture has been growing steadily, with the current production exceeding 3.5 million tons annually, of which the annual production of crucian carp in China exceeds 2.8 million tons [9]. Studies indicate that as the concentration of saline-alkaline in water increases, the diversity of freshwater fish species decreases [10]. However, crucian carp exhibits relatively strong tolerance in high-concentration saline-alkaline waters compared to other saline-alkaline-tolerant species such as bighead carp (*Luciobarbus capito*), Darli Lake Waleh Yarrow fish (*Leuciscus Waleckii*), and naked carp (*Kessler)* from Qinghai Lake [11]. Crucian carp, as a vertebrate, has evolved with multiple ploidy levels and the ability for both sexual and asexual reproduction [12]. Among them, compared with diploid crucian carp, triploid crucian carp has excellent characteristics such as wide feeding habits, a fast growth rate, and a high body condition coefficient [13]. Numerous publicly available reports detail the phenotypic and genetic breeding differences between triploid and diploid crucian carp. For instance, Cai [14] investigated the differences in muscle volatile components between diploid and triploid crucian carp, Liu [15] studied the biological traits of diploid and triploid crucian carp, and Li [16] utilized transcriptomics to analyze the differences in growth, reproduction and immune-related genes between diploid and triploid crucian carp. However, research specifically focusing on the differences in their saline-alkaline-tolerance traits has not been publicly reported to date.

In recent years, with the advancement of technology, techniques such as transcriptomics, proteomics, and metabolomics have found widespread application in the study of the stress response, ecotoxicology, and growth and development of aquatic organisms, becoming powerful tools for researching physiological changes and organism metabolism in aquatic life [17,18]. Transcription controls the translation expression of proteins, while protein activity reflects the organism’s fundamental physiological activities. Ultimately, the metabolic behavior at the cellular level can accurately reflect the terminal information of the organism [19]. Metabolomics, being capable of both qualitative and quantitative analysis of small-molecule metabolites within an organism, provides a novel research approach for elucidating the metabolic characteristics and mechanisms of fish under saline-alkaline stress. It offers a scientific basis for unraveling the molecular mechanisms underlying specific biological processes. Currently, there is limited research on the metabolic differences of diploid and triploid crucian carp under saline-alkaline stress. Publicly available scientific reports regarding the differential analysis of saline-alkaline tolerance traits at the cellular level are lacking, constraining the development of superior traits in saline-alkaline-tolerant fish.

Blood, as one of the crucial tissues in an animal’s body, plays vital physiological roles in energy transport, physiological regulation, and defense, and is closely associated with the animal’s growth and physiological state [20]. When fish face metabolic disturbances due to external environmental stressors, the metabolites in their blood also undergo changes [21]. In previous research conducted by our team using non-targeted metabolomics based on Ultra-performance liquid chromatography coupled with quadrupole time-of-flight mass spectrometry (UPLC-QTOF/MS), plasma and gills of the squarehead catfish and bighead carp were studied as target tissues and organs under NaHCO_3_ saline-alkaline stress. This research revealed widespread changes in the plasma metabolome under saline-alkaline stress, with cluster analysis indicating alterations in key metabolic pathways related to amino acid synthesis and metabolism, arachidonic acid metabolism, and ketone body metabolism [22,23,24]. However, there is currently limited research on the metabolic differences in the serum of diploid and triploid crucian carp under carbonate-alkaline stress. Hence, this study employed non-targeted metabolomics technology based on UPLC-QTOF/MS to analyze significant metabolic differences in the serum of diploid and triploid crucian carp under different NaHCO_3_ concentrations (0 mmol/L, 20 mmol/L, 60 mmol/L) during saline-alkaline stress. This research aimed to further elucidate the metabolic differences at the cellular metabolism level in diploid and triploid crucian carp under NaHCO_3_ stress, providing scientific theoretical support for the cultivation of superior saline-alkaline-tolerant fish varieties.

## 2. Materials and Methods

### 2.1. Experimental Materials

Sodium bicarbonate (NaHCO_3_, analytical grade, Tianjin Chemical No.3 Factory Co, Tianjin, China); Tricaine (MS-222, St. Louis, MO, USA); liquid nitrogen (Harbin Liming Gas Co., Ltd., Harbin, China); methanol (mass spectrometry grade, Merck & Co, Darmstadt, Germany); acetonitrile, formic acid (mass spectrometry grade, Merck & Co, Darmstadt, Germany); ammonia solution (mass spectrometry grade, Thermo Fisher Scientific, Pasadena, CA, USA).

### 2.2. Experimental Subjects

This study was conducted in strict accordance with Heilongjiang River Fisheries Research Institute of CAFS Application for Laboratory Animal Welfare and Ethical review (Figure S4). The crucian carp used in this experiment were procured from the Heilongjiang Fisheries Research Institute of the Chinese Academy of Fishery Sciences at the Harbin Experimental Station. Individuals with robust health, free from diseases or injuries, weighing (130.67 ± 7.19) grams, and measuring (17.83 ± 1.28) centimeters were selected. Each fish’s ploidy was determined using a flow cytometer. Based on the identification results, the fish were categorized into diploid crucian carp (2n with a cell chromosome count of 100) and triploid crucian carp (3n with a cell chromosome count of 150). The fish were separated into eight indoor recirculating aquaculture systems (dimensions: 100 cm × 45 cm × 50 cm) to start to acclimate. The experimental water was sourced from tap water aerated for 72 h. The water temperature was maintained at 24 °C to 26 °C. Freshwater control pH was about 7.1, ammonia <1.0 mg/L, and dissolved oxygen > 7.5 mg/L. A 20 mmol/L NaHCO_3_ stress group with a pH of about 8.4, ammonia < 1.0 mg/L, and dissolved oxygen > 7.5 mg/L was established. Finally, a 60 mmol/L NaHCO_3_ stress group with a pH of about 9.6, ammonia < 1.0 mg/L, and dissolved oxygen > 7.5 mg/L was established. The photoperiod was set at 12 to 14 h daily. During the acclimatization period, the fish were fed in a prescribed manner at scheduled intervals for two weeks.

### 2.3. Experimental Methods and Sample Collection

For this experiment, a total of 360 healthy individuals, including both 2n and 3n, were selected. The experiment comprised two freshwater control groups (Con-2n, Con-3n) and four NaHCO_3_ stress groups: 20 mmol/L (CA20-2n, CA20-3n), and 60 mmol/L (CA60-2n, CA60-3n). Each group had three replicates, with each replicate containing 20 individuals. To prevent acute stress reactions in 2n and 3n, the exposure concentrations were gradually increased at a rate of 5 mmol/L per day to reach the required concentration after four days, and then the exposure experiment was started for 30 days. During the experiment, water was changed every 10 days, with the water replacement volume being one-third of the tank volume. Sodium bicarbonate was appropriately replenished to maintain the experimental concentrations. Water quality parameters and NaHCO_3_ concentrations were monitored throughout the exposure experiment to ensure water stability.

Feeding was halted 24 h before sampling. Fifteen fish were randomly selected from each group and immersed in an anesthetic solution (MS-222) with a concentration of 40 mg/L. After deep anesthesia, 2 mL of blood was collected from the caudal vein. Subsequently, the blood samples were left to stand in a refrigerator at 4 °C for 12 h, followed by centrifugation (4 °C, 3500 r/min) for 10 min. The obtained serum was stored at −80 °C in an ultra-low temperature freezer for further analysis.

### 2.4. Metabolite Extraction

Frozen serum samples stored at −80 °C (100 μL) were thawed at 4 °C. To each sample, 400 μL of prechilled 80% methanol-water solution (4 °C) was added. The mixture was vortexed for 60 s, subjected to ultrasonication in an ice bath for 10 min, and then left to stand at −20 °C for 30 min. Subsequently, the samples were centrifuged at 13,000 r/min for 10 min at 4 °C, and 300 μL of the supernatant was filtered through a 0.22 μm organic membrane into the injection bottle for subsequent analysis. Simultaneously, for each serum sample, 50 μL was taken and mixed uniformly to prepare quality control (QC) samples using the aforementioned method [25]. These QC samples were utilized for assessing sample stability and instrument reliability.

### 2.5. UPLC-QTOF-MS Analysis

Column: BEH C18 column (50 mm × 2.1 mm, 1.7 μm; Waters, Milford, MA, USA); Column Temperature: 40 °C; Sample Injector Temperature: 4 °C; Injection Volume: 10 μL; Flow Rate: 0.30 mL/min; Positive Ion Scan: Mobile phase A: 0.1% formic acid in water; Mobile phase B: 0.1% formic acid in acetonitrile; Negative Ion Scan: Mobile phase A: 0.1% ammonia solution in water; Mobile phase B: 0.1% ammonia solution in acetonitrile; Gradient Elution Program: 0 to 4 min, 5% to 70% B; 4 to 11 min, 70% to 85% B; 11 to 12 min, 85% to 100% B; 12 to 13 min, 100% B; 13 to 13.2 min, 100% to 5% B; 13.2 to 15 min, 5% B.

Ion Source: Electrospray ionization source (ESI); Ion Source Voltage: Positive mode: 5500 V, Negative mode: −4500 V; Ion Source Temperature: 550 °C; Collision Voltage: Positive mode: 80 V, Negative mode: −80 V; Collision Energy: Positive mode: 35 eV, Negative mode: −35 eV; Collision Energy Spread: Positive mode: 15 eV, Negative mode: −15 eV; Nebulizing Gas (Gas1) Pressure: 55 PSI, Auxiliary Gas (Gas2) Pressure: 55 PSI; Curtain Gas (Cur) Pressure: 35 PSI; Dynamic Background Subtract (DBS) function enabled; Information-Dependent Acquisition (IDA) triggered, with the top 8 peaks exceeding 100 cps subjected to second-level mass spectrometry scan; First-level mass spectrometry ion scan range: 100–1200 Da; Second-level mass spectrometry ion scan range: 50–1000 Da.

### 2.6. Data Processing

The raw chromatographic peaks obtained from UPLC-QTOF/MS were imported into Progenesis QI 2.1 software (Waters Corporation, Milford, MA, USA) software for data preprocessing, resulting in a two-dimensional data matrix containing information such as mass-to-charge ratio, retention time, sample names, and peak areas. Subsequently, the generated two-dimensional data matrix was imported into SIMCA 14.0 software for Principal Component Analysis (PCA) and Orthogonal Partial Least Squares Discrimination Analysis (OPLS-DA) on each group’s data. This analysis revealed the discrete trends of metabolites between the control and treatment groups. Differential metabolites (DMs) were selected based on criteria such as Variable Importance in Projection (VIP > 1) and T-test analysis (*p* < 0.05). Further identification and classification of all DMs were conducted using the HMDB database. Finally, the selected DMs were imported into MetaboAnalyst 5.0 for metabolic pathway enrichment analysis.

## 3. Results

### 3.1. Metabolic Profiles

**Figure 1:**
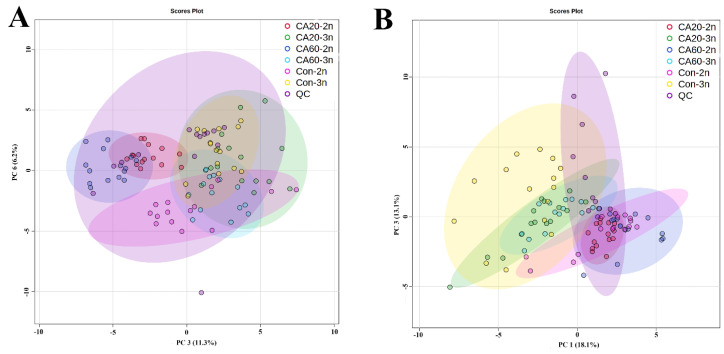
The PCA scores plots of 2n and 3n in different NaHCO_3_ concentration groups in positive ion modes (**A**); The PCA scores plots of 2n and 3n in different NaHCO_3_ concentration groups in negative ion modes (**B**); Con is freshwater control group, CA20 is the 20 mmol/L NaHCO_3_ exposure group, CA60 is the 60 mmol/L NaHCO_3_ exposure group, 2n is diploid crucian carp, 3n is triploid crucian carp.

**Figure 2:**
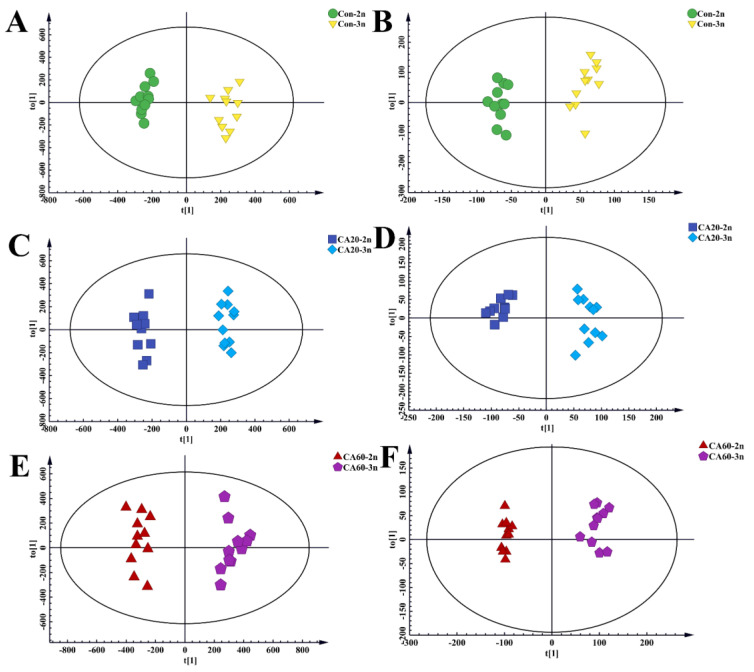
The OPLS-DA score plots of 2n and 3n in Con in positive ion modes (**A**), The OPLS-DA score plots of 2n and 3n in Con in negative ion modes (**B**); The OPLS-DA score plots of 2n and 3n in CA20 concentration in positive ion modes (**C**), The OPLS-DA score plots of 2n and 3n in CA20 concentration in negative ion modes (**D**); The OPLS-DA score plots of 2n and 3n in CA60 concentration in positive ion modes (**E**), The OPLS-DA score plots of 2n and 3n in CA60 concentration in negative ion modes (**F**).

The obtained data underwent PCA and OPLS-DA model analysis. PCA, an unsupervised classification model, is used to continuously reduce multidimensional data to several principal components that describe the characteristics of the original data. It is primarily employed to observe the overall distribution of samples. As shown in Figure 1, positive (Figure 1A) and negative (Figure 1B) ion modes exhibited a certain separation trend between 2n and 3n under different NaHCO_3_ concentration stress. Additionally, the distribution of QC samples showed good consistency, indicating that the instrument is in a stable state, and the obtained data are reliable. OPLS-DA, a supervised classification model, emphasizes differences between groups by maximizing the contrast between groups. The OPLS-DA score plots in Figure 2 for positive ions (Figure 2A,C,E) and negative ions (Figure 2B,D,F) showed that samples of 2n and 3n are completely distributed in different regions, indicating that different NaHCO_3_ concentrations have distinct effects on the serum of 2n and 3n.

### 3.2. Selection of Differentially Expressed Metabolites and Metabolic Pathway Analysis

**Figure 3:**
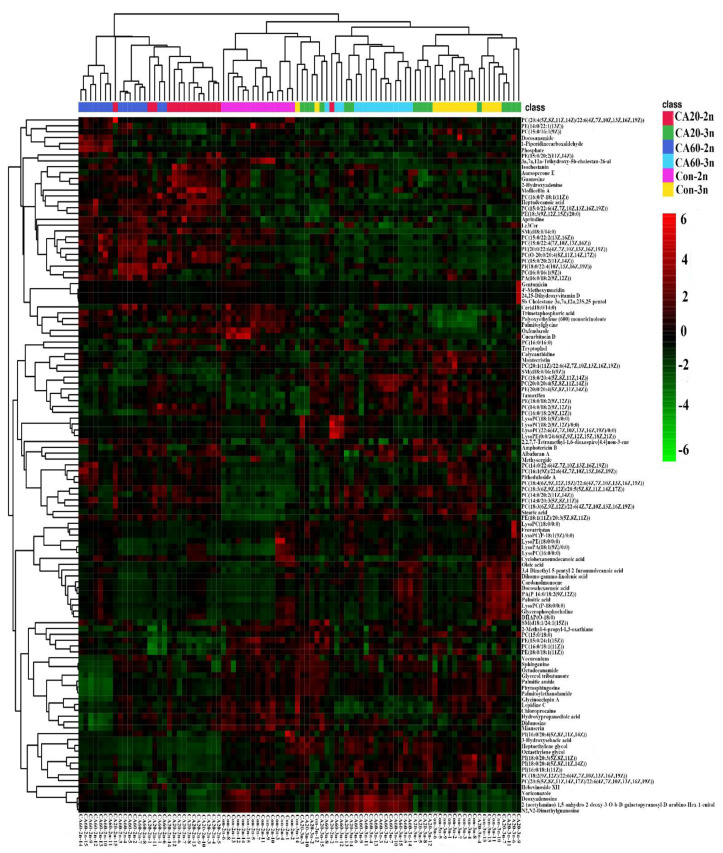
Cluster heatmap of DMs.

**Figure 4:**
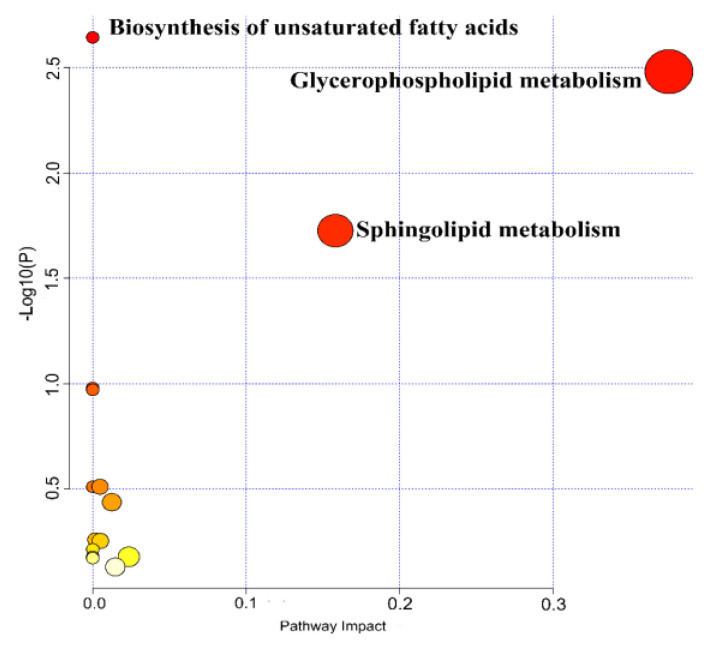
The bubble diagram of metabolic pathways.

Differentially metabolites (DMs) were screened based on criteria of VIP > 1 and *p* < 0.05, resulting in the identification of 119 DMs. Subsequently, cluster analysis was performed, as shown in Figure 3. The identified DMs were further validated using the HMDB database. In the Con-2n and Con-3n groups, 113 DMs were identified, while 69 DMs were common to the CA20-2n and CA20-3n groups, and 46 DMs were common to the CA60-2n and CA60-3n groups. These DMs belonged to various metabolite types, including glycerophospholipids, sphingolipids, purine nucleotides and derivatives, fatty acids, fatty acid amides, bile acids, inorganic phosphates, amino acids, and aromatic compounds, among others. To further elucidate the metabolic behavior differences between 2n and 3n under saline-alkaline stress, the identified DMs were subjected to metabolic pathway enrichment analysis using MetaboAnalyst 5.0 software, in which the most affected pathways were highlighted in red. The results indicated that DMs were mainly enriched in 15 metabolic pathways, including linoleic acid metabolism, glycerophospholipid metabolism, purine metabolism, sphingolipid metabolism, unsaturated fatty acid biosynthesis, ether lipid metabolism, GPI-anchor biosynthesis, α-linolenic acid metabolism, glycerolipid metabolism, inositol phosphate metabolism, arachidonic acid metabolism, fatty acid degradation, primary bile acid biosynthesis, fatty acid elongation, and fatty acid biosynthesis. Specifically, in both CA20 and CA60 groups, 2n and 3n exhibited significant correlations with three metabolic pathways: glycerophospholipid metabolism, unsaturated fatty acid biosynthesis, and sphingolipid metabolism (Figure 4).

## 4. Discussion

Saline-alkaline water resources are widely distributed in the land water system, and the rational development and utilization of saline water resources can not only effectively alleviate the problem of the serious shortage of freshwater resources but also promote the sustainable development of saline-alkaline aquaculture [26]. Previous studies [27] have indicated that in freshwater aquaculture environments, triploid (3n) individuals exhibit superior traits such as faster growth and stronger stress resistance compared to diploid (2n) individuals. However, the differences in stress resistance and growth between 2n and 3n in saline-alkaline water environments are not well understood. In this context, unraveling the metabolic differences at the cellular level between 2n and 3n in saline-alkaline environment was crucial. It held important implications for furthering precise targeted metabolic regulation, enhancing the efficiency of aquaculture in saline-alkaline water areas, and was considered an effective approach for the efficient utilization of saline-alkaline water resources. To elucidate the metabolic behavioral differences of 2n and 3n under saline-alkaline stress, this study conducted exposure experiments in an indoor NaHCO_3_ environment, focusing on metabolic profiling of the serum from 2n and 3n individuals. The results demonstrated that exposure to NaHCO_3_ at the same concentration significantly disrupted metabolic pathways in the serum, including glycerophospholipid metabolism, sphingolipid metabolism, and the biosynthesis of unsaturated fatty acids. This finding suggested that the stress response in terms of metabolic changes was conserved across both 2n and 3n individuals under saline-alkaline exposure. Understanding these metabolic alterations provides valuable insights for targeted metabolic regulation, contributing to improved aquaculture efficiency in saline-alkaline water environments. This study serves as a foundational step towards the efficient utilization of saline-alkaline water resources.

### 4.1. Glycerophospholipid Metabolism Pathway

The metabolomic results indicated that exposure to carbonate-saline-alkaline environments disrupts glycerophospholipid metabolism in the serum of both 2n and 3n individuals, leading to a destabilization of the serum. Lipids play a crucial role as a source of energy for organisms and contribute significantly to various physiological functions, including metabolism [28], protein recognition on cell membranes, and signal transduction [29]. Glycerophospholipids, such as phosphatidylcholine (PC), phosphatidylethanolamine (PE), and phosphatidic acid (PA), are essential components of cell membranes. Changes in their concentrations directly affect the composition and permeability of cell membranes, thereby influencing cellular processes like apoptosis [30], immune response [31], and inflammation [32]. PA [33], in particular, serves as a vital mediator in various lipid metabolic pathways. PC [34] and PE [35], major constituents of biological membranes, account for approximately 75% of the total phospholipid content in cells. They are essential to maintaining cell integrity and storing energy [36]. Additionally, they serve as substrates for generating various lysophospholipids, including lysophosphatidylcholine (LysoPC), lysophosphatidylethanolamine (LysoPE), and lysophosphatidic acid (LysoPA). The production of Lysophospholipids can lead to cell membrane damage or cell death [37].

Under external environmental stress, excess reactive oxygen species (ROS) bind to unsaturated fatty acids and cholesterol on cell membranes, causing lipid peroxidation, leading to reduced cell membrane fluidity and altered cellular function, which can cause irreversible damage to the organism [38]. The identified glycerophosphocholine (GPC) in this study, upregulated significantly in 3n but not significantly in 2n at CA20 concentration, indicated that 2n exhibits a favorable adaptive response to low carbonate saline-alkaline-concentration stress compared to 3n. However, both showed a downregulation trend in CA60, with 3n exhibiting a significantly higher degree of downregulation than 2n. This suggested that with increasing saline-alkaline concentration, the organism’s stress response surpassed its tolerance, exacerbating damage and compromising its ability to maintain GPC balance.

Furthermore, in CA20 and CA60 concentrations, there was a significant downregulation trend of Lysophospholipids (LysoPC(22:6(4Z, 7Z, 10Z, 13Z, 16Z, 19Z)/0:0), LysoPC (16:0/0:0), LysoPC (18:1(9Z)/0:0), LysoPA (18:1(9Z)/0:0), and LysoPE (18:0/0:0)) in 3n, while no significant changes were observed in 2n. These results collectively suggested that under high carbonate saline-alkaline stress, the rate of lipid peroxidation was faster in 3n than in 2n. The increase in phospholipids in 2n played a crucial role in maintaining cell integrity, indicating that 2n had the capability to ensure the normal physiological function of PC and PE substances. Simultaneously, at CA20 and CA60 concentrations, Lysophospholipids were significantly upregulated in 3n, indicating more severe destruction of the double-layer structure of the lipid membrane, higher levels of cell death, and subsequent inflammation. This reflected 3n’s poorer performance in saline-alkaline stress tolerance compared to 2n.

### 4.2. Sphingolipid Metabolism Pathway

Sphingolipids, as structural molecules in biological membranes [39], play a crucial role in maintaining the barrier function of cells and the fluidity of cell membranes. They are involved in regulating essential signal transduction processes, influencing cell growth, aging, and programmed cell death [40]. Sphingomyelins (SM), the most abundant type of sphingolipids, are essential lipids in cell membranes and plasma lipoproteins. They play a vital role in regulating various cell processes’ biological activities, such as proliferation, differentiation, apoptosis induction, and inflammation. SM is hydrolyzed by sphingomyelinase to produce a crucial product, ceramide (Cer). Cer, acting as a second messenger, can activate downstream effector molecules and further metabolize into sphingosine (Sph) and free fatty acids through ceramidase [41]. Studies have shown that Cer and Sph can effectively inhibit cell growth and induce apoptosis [42]. Palmitic acid (PA), the most abundant free fatty acid in the blood, is a major inducer of lipotoxicity. Research has indicated that PA can significantly induce cell damage, and the severity of cell apoptosis increases with its concentration [43].

In this study, at CA20 and CA60 concentrations, the content of SM [d18:1/18:1 (9Z)] was downregulated in both 2n and 3n, while SM [d18:1/24:1 (15Z)], PA, and Sph content were upregulated in both 2n and 3n. However, the changes in these DEMs were significantly higher in 3n than in 2n. This indicated that carbonate saline-alkaline stress disrupts sphingolipid metabolism in the crucian carp organism, specifically leading to the disturbance of sphingolipid metabolism related to inflammatory responses. The degree of inflammation response in 3n is significantly higher than in 2n, resulting in severe lipid metabolism disruption and inducing cell apoptosis.

Simultaneously, crucian carp produced a large amount of sphingomyelin through sphingolipid metabolism to resist damage caused by saline-alkaline environments. This finding is consistent with the metabolic study of crucian carp gills under saline-alkaline stress conditions [44].

### 4.3. Biosynthesis Metabolic Pathway of Polyunsaturated Fatty Acids

Unsaturated fatty acids can be classified into monounsaturated fatty acids and polyunsaturated fatty acids (PUFAs) based on the number of double bonds. Studies have shown that monounsaturated fatty acids such as oleic acid (OA) have a preventive and protective effect on lipotoxic metabolic abnormalities caused by saturated fatty acids [45]. PUFAs, as unique bioactive substances, play a crucial role in antioxidant [46], anti-inflammatory [47], and immune regulation [48]. According to the position and function of double bonds, PUFAs can be further divided into ω-3, ω-6, ω-9, etc. [49]. In PUFA molecules, the first unsaturation appears at the third position from the methyl end of the carbon chain, called ω-3 PUFA, mainly including alpha-linolenic acid (ALA), eicosapentaenoic acid (EPA), and docosahexaenoic acid (DHA). Research has shown that ω-3 PUFAs can improve inflammatory responses. When the repair function of the body is stronger than the occurrence of inflammation, the body can maintain normal physiological activities. Conversely, when the repair function cannot compensate for inflammation damage, the body tends to physiological imbalance, and prolonged imbalance can lead to irreversible physical damage or even death. DHA plays an important role in enhancing the body’s immune system [50].

In the PUFA molecule, ω-6 PUFA is typically characterized by its farthest double bond from the carboxyl group on the penultimate sixth carbon atom, mainly including linoleic acid, gamma-linolenic acid, and arachidonic acid (AA), among others [51]. Linoleic acid, as an essential fatty acid in the body, is converted into gamma-linolenic acid under the action of the rate-limiting enzyme Δ6 desaturase, which is finally upgraded to dihomo-γ-linolenic acid, and then generates arachidonic acid by the successive action of an elongase and a D5 desaturase [52]. Arachidonic acid, as an inflammatory signaling factor in the biosynthesis metabolic pathway of unsaturated fatty acids, can improve oxidative stress reactions and regulate lipid metabolism [53]. Some studies have shown that ω-3 PUFAs and ω-6 PUFAs have a competitive inhibitory relationship. Therefore, an imbalance in the ω-6/ω-3 PUFAs ratio in the body can cause confusion in various physiological functions and aggravate lipid metabolism disorders [54]. Moreover, an excessively high content of ω-3 PUFAs can inhibit immune function and increase lipid oxidation [55].

The results of the screened DEMs in this study showed that, in the CA20 and CA60 groups, the content of dihomo-γ-linolenic acid (DGLA) in 3n serum was upregulated, while it was downregulated in 2n. This suggested that oxidative stress reactions were more severe in 3n than in 2n under saline-alkaline stress, leading to the disturbance of biosynthesis metabolic pathways of unsaturated fatty acids involving DHA and triggering inflammation and cell apoptosis in crucian carp cells. At the same time, in the CA20 and CA60 groups, the significantly altered metabolites dihydroxyacetone phosphate (DHAP) and oleic acid (OA) in 3n were significantly upregulated, while they were significantly downregulated in 2n. This indicated that under saline-alkaline stress, the increase in the content of DHA and OA in 3n was excessive, inhibiting the body’s immune function and causing an imbalance in the body’s immune and antioxidant systems, resulting in aggravated lipid metabolism disorders. Therefore, under carbonate saline-alkaline stress, the inflammatory symptoms of 3n were significantly more severe than those of 2n.

## 5. Conclusions

By employing non-targeted metabolomics using UPLC-QTOF/MS, we conducted a comprehensive analysis of the serum metabolome of 2n and 3n crucian carp under saline-alkaline stress. The results revealed significant alterations in various compound classes, including glycerophospholipids, sphingolipids, purine nucleotides and derivatives, and amino acids, under stress conditions. Further statistical analysis indicated that the differentially expressed metabolites were primarily associated with glycerophospholipid metabolism, sphingolipid metabolism, and the biosynthesis of unsaturated fatty acids. Specifically, the disturbance in serum sphingolipid metabolism suggested that both 2n and 3n experienced toxicity under NaHCO_3_ stress, with 3n displaying greater sensitivity to saline-alkaline stress, evidenced by its weaker capacity to regulate oxidative stress and inflammatory responses, resulting in reduced tolerance to external saline-alkaline environments. Conversely, pathways related to glycerophospholipid metabolism and the biosynthesis of unsaturated fatty acids appeared to mitigate the organismal damage caused by saline-alkaline stress. Consequently, in saline-alkaline environments, 2n was deemed more suitable for aquaculture activities compared to 3n.

This study provides a preliminary insight into the metabolic differences between 2n and 3n crucian carp at the cellular level under saline-alkaline stress, offering a theoretical foundation for understanding the regulatory mechanisms of 2n and 3n crucian carp in adapting to saline-alkaline environments.

## Data Availability

The data presented in this study are available on request from the corresponding author due to privacy.

## Supplementary Materials

The following supporting information can be downloaded at: https://www.mdpi.com/article/10.3390/metabo14120721/s1, Table S1: The data matrix of positive ion model for PCA and OPLS-DA analysis. Table S2: The identified significantly different metabolites between diploid crucian carp and triploid crucian carp. Figure S1: Cross-validation plot of 2n-20 VS 2n-60 blood samples. Figure S2: Cross-validation plot of 3n-20 VS 3n-60 blood samples. Figure S3: Map of saline-alkaline areas in China. Figure S4: Heilongjiang River Fisheries Research Institute of CAFS Application for Laboratory Animal Welfare and Ethical review.
