# Supplementary material for: Analysis of Immunosuppression and Antioxidant Damage in Diploid and Triploid Crucian Carp (Carassius auratus) Induced by Saline-Alkaline Environmental Stress: From Metabolomic Insight
Source: Metabolites. 2024 Dec 21;14(12):721. doi: 10.3390/metabo14120721 (PMC11677042; doi:10.3390/metabo14120721)
Supplement: Supplementary file 1 [file metabolites-14-00721-s001.zip › S4.pdf]

黑龙江水产研究所实验动物福利伦理审查申请书

Heilongjiang River Fisheries Research Institute of CAFS

Application for Laboratory Animal Welfare and Ethical review

申请编号 Issue No.: 20210316-001　　申请日期 Appl. Date: 2021.3.16

申请原因：项目申报 Program application☐　　动物实验 Animal experiment☑

项目名称 Program	（项目申报时填写）		
实验名称 Program	（动物实验时填写）碳酸盐碱环境对鲫的胁迫作用机制		
项目起止日期 Program period	年　月　日至　年　月　日		
动物实验日期 Experimental period	2021 年 4 月 12 日 至 2023 年 12 月 20 日		
项目负责人 PI	孙言春	实验执行人 Director of animal experiment	耿传业，刘文质，韩琳，袁芳英等

实验动物概况 Overview of Lab. Animals	动物来源 Animal origin	哈尔滨市呼兰区/Hulan Dist. of Harbin	年龄或体重 Age or Weight	2 龄，(130.67 ± 7.19)g
	品种/品系 Breed/Strain	黑龙江鲫	数量 Number	540

实验要点（包括实验方法、观测指标、实验结束后处死动物的方法等/Experimental methods, observation indexes, and methods of killing animals after the experiment）

实验用鲫采集自黑龙江水产研究所呼兰试验站，在室内循环池中饲养两周。MS-222 麻醉后采集全血、肝胰脏、鳃、胆囊、心脏、肾脏、脾脏、肠道等组织，开展代谢组学、转录组学、生理生化指标等测试。采集完样本后，动物残骸按照黑龙江水产研究所废弃物品管理条例处理。

The experimental crucian carp were from the Hulan Experimental Station of the Heilongjiang Fisheries Research Institute and raised in an indoor circulating pool for two weeks. Collect whole blood, liver and pancreas, gills, gallbladder, heart, kidney, spleen, intestine and other tissues after MS-222 anesthesia, and conduct metabolomics, transcriptomics, physiological and biochemical indicators testing. After collecting the samples, the animal remains were disposed of in accordance with the regulations on waste management of Heilongjiang Fisheries Research Institute.

声明 Declaration

我将严格遵守实验动物福利伦理原则，随时接受黑龙江水产研究所实验动物福利伦理委员会的监督与检查。

I will abide by the ethical principles of laboratory animal welfare, and accept the supervision and inspection by the committee of Heilongjiang Fisheries Research Institute.

项目负责人 PI：[illegible]

实验执行人：刘文质. 韩琳. 耿佳业, 袁芳英

Director of animal experiment

2021年 3 月 16 日

审查意见

同意□✓　　　　不同意□

Approval　　　　Disapproval

委员会主任签名：[illegible]

Authorized Personnel Signature

中国水产科学研究院黑龙江水产研究所
科研处

公章：

2021年 3 月 16 日
