# Supplementary figures and images for: Analysis of Immunosuppression and Antioxidant Damage in Diploid and Triploid Crucian Carp (Carassius auratus) Induced by Saline-Alkaline Environmental Stress: From Metabolomic Insight
Source: Metabolites. 2024 Dec 21;14(12):721. doi: 10.3390/metabo14120721 (PMC11677042; doi:10.3390/metabo14120721)

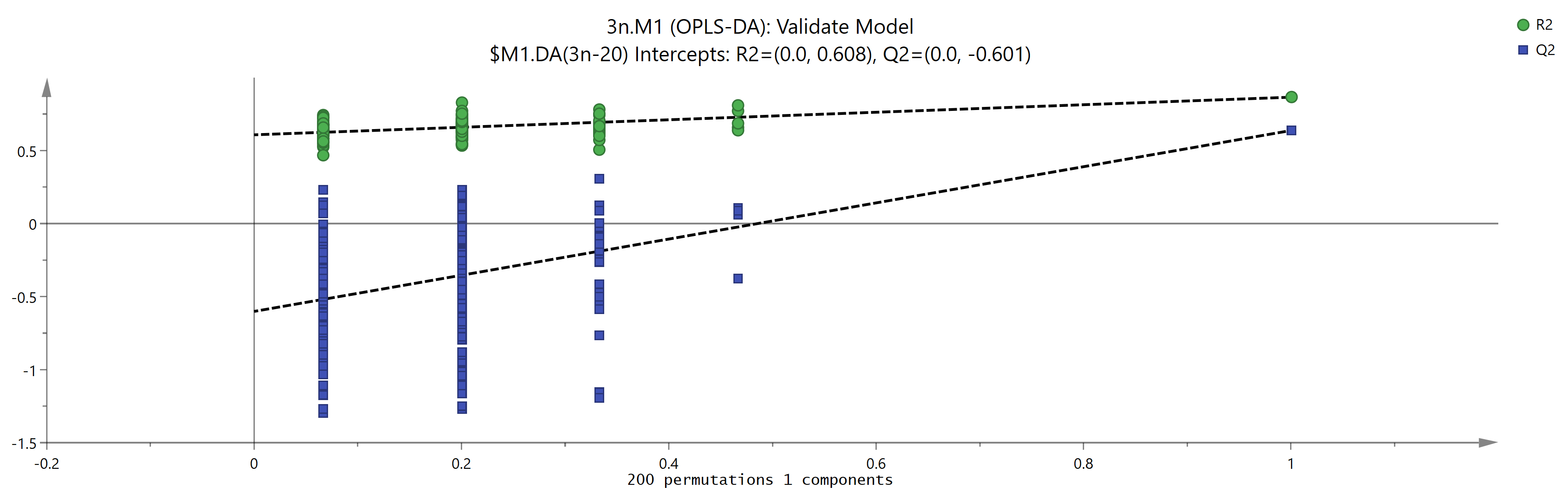

Supplement: Supplementary file 1 [file metabolites-14-00721-s001.zip › Figure S2.png]

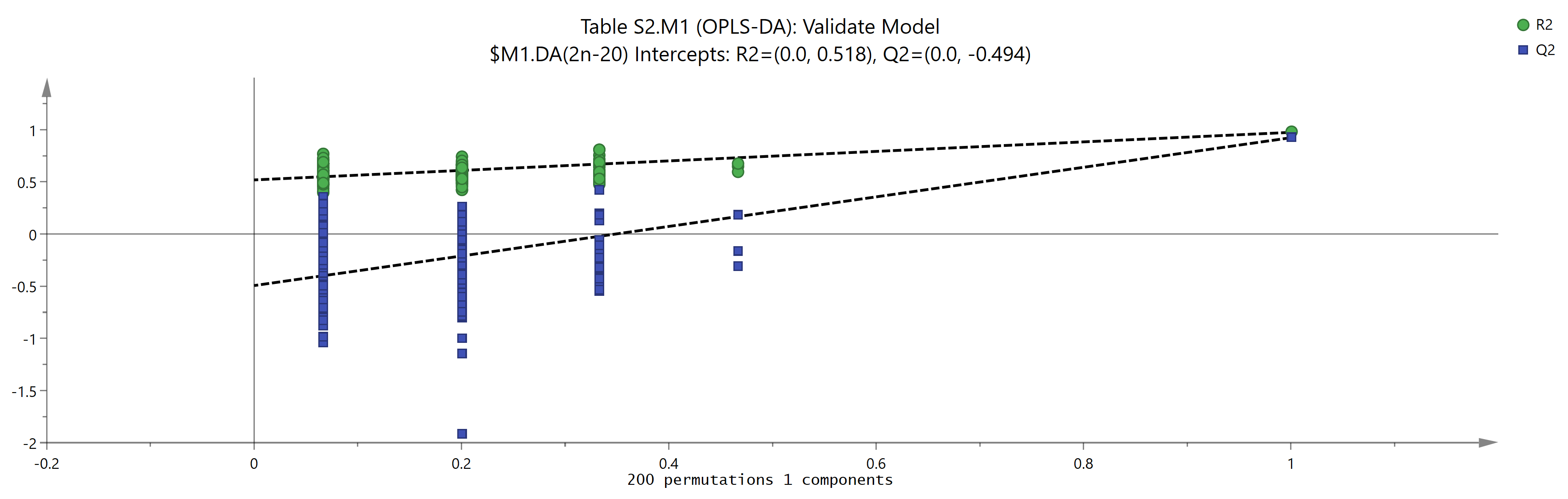

Supplement: Supplementary file 1 [file metabolites-14-00721-s001.zip › Figure S1.png]
